# A Sensitive and Specific Competitive Enzyme-Linked Immunosorbent Assay for Serodiagnosis of COVID-19 in Animals

**DOI:** 10.3390/microorganisms9051019

**Published:** 2021-05-10

**Authors:** Susanna K. P. Lau, Zirong He, Chi-Ching Tsang, Tony T. Y. Chan, Hayes K. H. Luk, Elaine Chan, Kenneth S. M. Li, Joshua Fung, Franklin W. N. Chow, Anthony R. Tam, Tom W. H. Chung, Sally C. Y. Wong, Tak-Lun Que, Kitty S. C. Fung, David C. Lung, Alan K. L. Wu, Ivan F. N. Hung, Jade L. L. Teng, Ulrich Wernery, Suk-Wai Hui, Paolo Martelli, Patrick C. Y. Woo

**Affiliations:** 1 Department of Microbiology, Li Ka Shing Faculty of Medicine, The University of Hong Kong, Pokfulam, Hong Kong; hezirong725@hotmail.com (Z.H.); microbioct@connect.hku.hk (C.-C.T.); tonytychan1@gmail.com (T.T.Y.C.); hayesluk@gmail.com (H.K.H.L.); elaineee@gmail.com (E.C.); keth105@gmail.com (K.S.M.L.); mbjfung@connect.hku.hk (J.F.); cwn5810@gmail.com (F.W.N.C.); cwh366@ha.org.hk (T.W.H.C.); llteng@hku.hk (J.L.L.T.); 2 Department of Medicine, Queen Mary Hospital, Pokfulam, Hong Kong; antamwf@connect.hku.hk; 3 Department of Pathology, Queen Elizabeth Hospital, King’s Park, Hong Kong; wcy288@ha.org.hk (S.C.Y.W.); h9910303@yahoo.com.hk (D.C.L.); 4 Department of Pathology, Tuen Mun Hospital, Tuen Mun, Hong Kong; quetl@ha.org.hk; 5 Department of Pathology, United Christian Hospital, Kwun Tong, Hong Kong; fungsck@ha.org.hk; 6 Department of Clinical Pathology, Pamela Youde Nethersole Eastern Hospital, Chai Wan, Hong Kong; alanklwu@yahoo.com; 7 Department of Medicine, Li Ka Shing Faculty of Medicine, The University of Hong Kong, Pokfulam, Hong Kong; ivanhung@hku.hk; 8 Central Veterinary Research Laboratory, P.O. Box 597, Dubai, United Arab Emirates; cvrl@cvrl.ae; 9 Ocean Park Corporation, Aberdeen, Hong Kong; hui.suk.wai@oceanpark.com.hk (S.-W.H.); paolo.martelli@oceanpark.com.hk (P.M.)

**Keywords:** SARS-CoV-2, nucleocapsid protein, competitive ELISA, antibody, COVID-19

## Abstract

In addition to human cases, cases of COVID-19 in captive animals and pets are increasingly reported. This raises the concern for two-way COVID-19 transmission between humans and animals. Here, we developed a SARS-CoV-2 nucleocapsid protein-based competitive enzyme-linked immunosorbent assay (cELISA) for serodiagnosis of COVID-19 which can theoretically be used in virtually all kinds of animals. We used 187 serum samples from patients with/without COVID-19, laboratory animals immunized with inactive SARS-CoV-2 virions, COVID-19-negative animals, and animals seropositive to other betacoronaviruses. A cut-off percent inhibition value of 22.345% was determined and the analytical sensitivity and specificity were found to be 1:64–1:256 and 93.9%, respectively. Evaluation on its diagnostic performance using 155 serum samples from COVID-19-negative animals and COVID-19 human patients showed a diagnostic sensitivity and specificity of 80.8% and 100%, respectively. The cELISA can be incorporated into routine blood testing of farmed/captive animals for COVID-19 surveillance.

## 1. Introduction

The COVID-19 pandemic has already officially infected more than 152 million patients with nearly 3,200,000 deaths worldwide as of 3 May 2021 [1]. The cause of it was confirmed to be a virus of the species *Severe acute respiratory syndrome–related coronavirus* (SARSr-CoV), named severe acute respiratory syndrome coronavirus 2 (SARS-CoV-2). So far, almost all cases were assumed to be due to human-to-human transmission. Since SARS-CoV-2 is able to infect a wide variety of cell lines originating from different animals [2], and it can also infect a number of animal species including ferrets, hamsters, macaques, mice, monkeys, rabbits, and tree shrews, in laboratory settings [3,4], it is important to understand the possibility of it causing natural infections in animals. Cases of SARS-CoV-2 infections in captive animals and pets, including dogs, domestic cats, and large felines such as tigers, lions, snow leopards, pumas and cougars, ferrets, minks, as well as gorillas were reported in the following places: Hong Kong, Belgium, China, the USA, the Netherlands, France, Spain, Germany, Russia, Denmark, the UK, Japan, South Africa, Italy, Sweden, Chile, Canada, Brazil, Greece, Argentina, Lithuania, Switzerland, Mexico, Slovenia, Estonia, Bosnia and Herzegovina, Poland, and Latvia [5]. In Valencia, Spain, around 1.5% of companion ferrets were found to be seropositive to SARS-CoV-2 in 2020 [6]. While a serological survey in Lombardy, Italy showed that only around 1% of free-ranging stray colony and abandoned shelter domestic cats in the region were seropositive to SARS-CoV-2 during the pandemic [7], another study in Wuhan, China demonstrated that the seroprevalence of SARS-CoV-2 in domestic cats from animal shelters, pet hospitals, or COVID-19 patients’ families could be as high as 14.7% [8]. Moreover, the possibility of SARS-CoV-2 transmission among domestic cats was also demonstrated in laboratory settings [9,10]. In addition, large scale outbreaks were reported in mink farms in Europe and North America [5]. In particular, at least 15,000 farmed minks died of COVID-19 in Michigan, Utah and Wisconsin in the USA [11]; and a wild mink in Utah was found to be positive for the disease [12]. Around 17 million farmed minks were culled in Denmark to prevent viral spreading [13]. In addition, worryingly, genomic studies in the Netherlands showed evidence of two-way COVID-19 transmission between humans and minks [14]. Therefore, there is a need to identify COVID-19 infections in animals, which would be undeniably important in the subsequent establishment of preventive measures.

The most widely used method for laboratory diagnosis of COVID-19 is quantitative reverse transcription–polymerase chain reaction (qRT–PCR). However, qRT–PCR can only detect the infection in its acute stage. As infections in animals can easily go unnoticed because they do not complain of fever, sore throat, etc., infections in the acute stage may be missed. Therefore, antibody detection is an excellent way of confirming the disease even after the animal has recovered from the illness. Since SARS-CoV-2 can infect a variety of animals, the optimal serological test would be one which is able to detect the SARS-CoV-2 antibody from different kinds of animal species. Currently a few in-house-developed or commercial multi-species indirect enzyme-linked immunosorbent assays (ELISAs) are available [15,16], where detection of the host antibodies relies on the use of a multi-species secondary antibody which recognises mammalian immunoglobulin G (IgG); however, utility of this multi-species secondary antibody has not yet been validated for all mammalian species [17]. In this study, we used the recombinant nucleocapsid (N) protein of SARS-CoV-2 and a monoclonal antibody (mAb) against it to develop a competitive ELISA (cELISA) for SARS-CoV-2 infection in virtually all kinds of animals. The assay developed was then validated for its analytical performance using positive serum samples from a laboratory guinea pig (*Cavia porcellus*) immunized with inactive SARS-CoV-2 virions and a human COVID-19 patient, as well as negative serum samples from dromedaries (*Camelus dromedarius*), Leschenault’s rousettes (*Rousettus leschenaulti*), and laboratory rabbits (*Oryctolagus cuniculus*) seropositive to other betacoronaviruses (βCoVs). The cELISA was further evaluated for its diagnostic sensitivity and specificity using serum samples from human qRT–PCR-confirmed COVID-19 patients as well as COVID-19-free animals from a local amusement park.

## 2. Materials and Methods

### 2.1. Ethics Statements

The use of experimental guinea pigs and rabbits in this study for antiserum production was approved by the Committee on the Use of Live Animals in Teaching and Research (CULATR), the University of Hong Kong (CULATR 5421-20). The use of leftover clinical specimens, such as human sera, in this study was approved by the Institutional Review Board of the University of Hong Kong/Hospital Authority Hong Kong West Cluster (UW 16-365).

### 2.2. Experimental Production of Anti-SARS-CoV-2 Antisera by Laboratory Animal Immunization

#### 2.2.1. Viral Strain and Culture

All experiments involving live SARS-CoV-2 virions were performed according to the approved standard operating procedures in our biosafety level 3 facility. SARS-CoV-2 strain HK20 (DDBJ/ENA/GenBank accession number MT186683), isolated from a laboratory confirmed COVID-19 patient in Hong Kong in February 2020 [18], was propagated using Vero E6 cells (American Type Culture Collection [ATCC], USA) in Dulbecco’s modified eagle medium (DMEM; Gibco, Grand Island, NY, USA) supplemented with 10% foetal bovine serum (*v*/*v*; Gibco) at 37 °C with 5% CO_2_. Cell culture supernatant was then harvested and stored at −80 °C until use. Viral titre was determined and expressed as the median tissue culture infectious dose (TCID_50_).

#### 2.2.2. Virus Inactivation and Purification

The harvested cell culture supernatant containing SARS-CoV-2 strain HK20 was centrifuged at 10,000× *g* for 20 min at 4 °C to remove cellular debris. The clarified supernatant was then inactivated with formaldehyde at a final concentration of 0.4% (*v*/*v*) at 37 °C for 5 days. The inactivated supernatant was then tested for viral viability by infecting Vero E6 cells at a 10-fold serial dilution with DMEM from 10^−1^ to 10^−5^, using live SARS-CoV-2 strain HK20 virus as a control. The cytopathic effect was checked daily, and the supernatant of each tissue culture well was collected on day 5, followed by the determination of the viral RNA copy number using the 2019 Novel Coronavirus RT–PCR Diagnostic Panel (Center for Disease Control and Prevention, Atlanta, GA, USA) according to the manufacturer’s protocol. The inactivated virus was then ultracentrifuged at 28,000× *g* overnight at 4 °C. Subsequently, the supernatant was discarded, and the virion pellet was washed twice with sterile phosphate-buffered saline (PBS; Gibco). The concentrated inactive virus was resuspended using 10% polyethylene glycol (PEG)-8000 (*w*/*v*; Sigma Aldrich, St. Louis, MO, USA) with 2.2% NaCl (*w*/*v*, Sigma Aldrich) and gently swirled at 4 °C overnight. The mixture was then centrifuged at 10,000× *g* for 30 min and the PEG-precipitated virions were resuspended with ice-cold sterile PBS. Resuspended virions were then added to a 40–60% sucrose gradient (*w*/*v* in 0.22 µm filter-sterilized PBS) drop by drop, followed by ultracentrifugation at 28,000× *g* for 2 h at 4 °C. The virion-containing fraction was then collected and washed by sterile PBS twice with ultracentrifugation at 28,000× *g* for 2 h at 4 °C to remove any residual sucrose. Lastly, the virion pellet was resuspended in PBS and viral concentration was determined using the Pierce BCA Protein Assay Kit (Thermo Scientific, Waltham, MA, USA).

#### 2.2.3. Animal Immunization

Ten guinea pigs and six rabbits aged 4–8 weeks old were obtained from the Centre for Comparative Medicine Research, the University of Hong Kong. All animals were each immunized with 1 μg of purified formaldehyde-inactivated SARS-CoV-2 strain HK20 virions in 30 μL of PBS mixed with an equal volume of the Alhydrogel adjuvant (2%; Invivogen, San Diego, CA, USA) via intramuscular injection in their legs. A booster dose was administered to the animals at 6 weeks post-immunization using the same dosage and injection procedures. Blood samples from each animal were collected prior to immunization (week 0), prior to booster administration (6 weeks post-immunization), as well as at 12, 16, and 20 weeks post-immunization.

#### 2.2.4. Antibody Detection

The titre of serum IgG against the SARS-CoV-2 N protein for each immunized animal was determined using a custom indirect ELISA. Briefly, each well of the microtitre plate (Thermo Scientific) was coated with 125 ng of the recombinant SARS-CoV-2 N protein (Sino Biological, Beijing, China) in 0.05 M carbonate buffer (15 mM Na_2_CO_3_ and 3.5 mM NaHCO_3_) per well at 4 °C overnight. After washing by PBS supplemented with 0.5% Tween 20 (*v*/*v*; PBST), and blocking with blocking buffer (10 mM of Tris, 0.2% gelatin [*w*/*v*], 2% sucrose [*w*/*v*], 0.02% thimerosal [*w*/*v*], 0.25% casein [*w*/*v*], and 0.5% Tween 20 [*v*/*v*]) for 2 h, each serum sample from the immunized animals, diluted in sample dilution buffer (1× PBS, 1% bovine serum albumin [BSA; *w*/*v*], and 0.1% Tween 20 [*v*/*v*]) at 100-fold, was added to one of the wells and then incubated at 37 °C for 1 h. After washing, the secondary antibody (goat anti-guinea pig IgG or goat anti-rabbit IgG) conjugated with horseradish peroxidase (HRP; Invitrogen, Carlsbad, CA, USA) diluted in the enzyme dilution buffer (1× PBS, 20% normal goat serum [*v*/*v*] and 0.1% Tween 20 [*v*/*v*]) at 2000-fold was added to the wells. Then, the microtitre plate was incubated at 37 °C for 30 min and washed again using PBS with 0.3% Tween 20 (*v*/*v*). An enzymatic reaction, using 100 µL of tetramethylbenzidine (TMB) solution (Life Technologies, Carlsbad, CA, USA) as the substrate, was developed for 10 min at room temperature and then stopped with 100 µL of 0.3 M H_2_SO_4_. The optical densities of the resultant reaction mixtures were read at 450 nm using the VICTOR X3 Multilabel Plate Reader (PerkinElmer, Waltham, MA, USA).

### 2.3. Serum Samples

#### 2.3.1. Sera for Initial Assay Development and Analytical Sensitivity Determination (Panel 1)

Four serum samples were collected for initial assay development and the determination of analytical sensitivity. One human serum sample (HK59-D20) from a laboratory confirmed COVID-19 patient which also tested seropositive to SARS-CoV-2 by the SARS-CoV-2 ELISA kit (Euroimmun, Lübeck, Germany), and one guinea pig serum sample from an experimentally immunized guinea pig (A) collected 6 weeks post-immunization as described above were included as positive sera. On the other hand, one human serum sample collected before the COVID-19 outbreak, and one healthy, untreated guinea pig serum sample, retrieved from our laboratory collection, were included as negative sera.

#### 2.3.2. COVID-19 Negative Sera for Cut-Off Determination (Panel 2)

A total of 74 COVID-19 negative sera were collected for cut-off determination. This included 9 guinea pig (B–J) and 6 rabbit (A–F) serum samples collected from the above experimental animals prior to experimental immunization (Panel 2a), as well as 59 other serum samples collected prior to the current COVID-19 outbreak and retrieved from our laboratory collection (Panel 2b). These consisted of 9 human sera obtained from Queen Mary Hospital, Hong Kong, 10 other guinea pig sera from our animal laboratory, 20 dromedary sera obtained from the Central Veterinary Research Laboratory (CVRL), Dubai, UAE, and 20 pig (*Sus scrofa domesticus*) sera obtained from the Veterinary Public Health Section, Food and Environmental Hygiene Department, the Government of the Hong Kong Special Administrative Region, Hong Kong. The two negative sera from Panel 1 were not included here.

#### 2.3.3. Sera from Animals Immunized with Inactive SARS-CoV-2 Virion (Panel 3)

A total of 60 sera, including 36 guinea pig and 24 rabbit serum samples, were collected from the above experimental animals (guinea pigs B–J and rabbits A–F) at 6, 12, 16, and 20 weeks post-inactive SARS-CoV-2 virion immunization. Serum samples from guinea pig A were not included here.

#### 2.3.4. Sera from Animals Infected by βCoVs for Determination of Analytical Specificity (Panel 4)

A total of 49 serum samples from animals infected with βCoVs of subgenera other than *Sarbecovirus*, which houses SARS-CoV-2, were retrieved from our laboratory collection. These included 12 dromedary serum samples seropositive to *Middle East respiratory syndrome-related coronavirus* (MERS-CoV, subgenus *Merbecovirus*), 9 dromedary serum samples seropositive to dromedary camel coronavirus UAE-HKU23 (DcCoV UAE-HKU23, subgenus *Embecovirus*) [19], 6 dromedary serum samples seropositive to both MERS-CoV and DcCoV UAE-HKU23 [19], 17 Leschenault’s rousette serum samples seropositive to *Rousettus bat coronavirus HKU9* (Ro-BatCoV HKU9, subgenus *Nobecovirus*) [20], as well as 5 serum samples from 5 laboratory rabbits experimentally infected with rabbit coronavirus HKU14 (RbCoV HKU14, subgenus *Embecovirus* [21]; collected on day 21 post-infection).

#### 2.3.5. Sera from Animals in an Amusement Park for Determination of Diagnostic Specificity (Panel 5)

Serum samples from 94 amusement park animals free from COVID-19, including dolphins (*Delphinus*; *n* = 63), giant pandas (*Ailuropoda melanoleuca*; *n* = 10), harbour seals (*Phoca vitulina*; *n* = 4), a koala (*Phascolarctos cinereus*; *n* = 1), spotted seals (*Phoca largha*; *n* = 6), sea lions (*Otariinae*; *n* = 5), and walruses (*Odobenus rosmarus*; *n* = 5), were obtained from the Ocean Park Corporation, Hong Kong.

#### 2.3.6. Sera from Laboratory Confirmed Human COVID-19 Patients for Comparison with Commercial Indirect ELISA and Determination of Diagnostic Sensitivity (Panel 6)

A total of 61 human serum samples from 61 COVID-19 patients (laboratory confirmed by qRT–PCR) were collected since January 2020 when the COVID-19 outbreak first appeared in Hong Kong. Among these 61 samples, 35 were obtained within 7 days post-qRT–PCR confirmation, 12 were obtained after 7 days post-qRT–PCR confirmation, and 14 were undated. The level of anti-SARS-CoV-2 antibodies in these human serum samples was tested using the SARS-CoV-2 ELISA kit (Euroimmun) following the manufacturer’s protocol. The positive human serum sample from Panel 1 (HK59-D20) was not included here.

### 2.4. Development of the cELISA

#### 2.4.1. Reactivity and Specificity of Recombinant SARS-CoV-2 N Protein with Mouse Anti-SARS-CoV-2 N Protein mAb

The reactivity of the recombinant SARS-CoV-2 N protein with the mouse anti-SARS-CoV-2 N protein mAb clone #05 (Sino Biological) was determined by Western blotting, using the inactive SARS-CoV-2 virions purified as described above as a control. Briefly, 40 ng of the recombinant SARS-CoV-2 N protein and 1 µg of inactive SARS-CoV-2 virion were first subjected to sodium dodecyl sulphate-polyacrylamide gel electrophoresis (SDS-PAGE) under a reducing condition. Then, the proteins were transferred onto an Immobilon-FL polyvinylidene fluoride membrane (Merck, Darmstadt, Germany) using the Trans-Blot SD Semi-Dry Transfer Cell (Bio-Rad, Hercules, CA, USA) under a voltage of 25 V for 40 min. The membrane was then blocked with 5% skimmed milk (Maxigenes, Dural, Australia; in PBST) at 4 °C overnight and subsequently incubated with 0.232 µg/mL of the mouse anti-SARS-CoV-2 N protein mAb at room temperature for 1 h. After washing with 0.3% PBST, the membrane was incubated with 1:4000 goat anti-mouse IgG secondary antibody conjugated with HRP (Invitrogen) in 0.3% PBST as a secondary antibody. Signal was developed using the WesternBright Quantum HRP substrate (Advansta, Menlo Park, CA, USA) and captured using the gel doc system G:BOX Chemi XRQ with the GeneSys software (Syngene, Cambridge, UK).

The specificity of the mouse anti-SARS-CoV-2 N protein mAb to the SARS-CoV-2 N protein was determined using an indirect ELISA. Briefly, wells of microtitre plates were coated with 0 to 62.5 ng of the recombinant SARS-CoV-2 N protein in a 0.05 M carbonate buffer at 4 °C overnight and then blocked using a blocking buffer at 37 °C for 2 h. The mouse anti-SARS-CoV-2 N protein mAb, serially two-fold diluted using a sample dilution buffer to the concentrations of 1.16 ng/µL to 0.145 ng/µL, was then added to each of the coated wells, followed by a 1 h incubation at 37 °C. Subsequently, the plates were incubated at 37 °C for 30 min with 1:1000 goat anti-mouse IgG secondary antibody conjugated with HRP. After washing, an enzymatic reaction was developed and stopped, and the optical densities of the resultant reaction mixtures were read as described above. The optimal concentrations of the recombinant SARS-CoV-2 N protein and mouse anti-SARS-CoV-2 N protein mAb for use in the subsequent development of the cELISA were determined as the antigen/antibody combination yielding the highest optical density.

#### 2.4.2. Evaluation of Epitope Blocking by mAb and Determination of Optimal Serum Dilution for the cELISA

Epitope blocking by the mouse anti-SARS-CoV-2 N protein mAb was evaluated and the optimal serum dilution for the cELISA was determined using sera from Panel 1 as described above. Briefly, the four serum samples were first two-fold serially diluted from 1:10 to 1:320 in a sample dilution buffer, and then 50 μL of each diluted serum sample were added to an independent well of the microtitre plate coated with the optimal amount of recombinant SARS-CoV-2 N protein. At the same time, 50 μL of mouse anti-SARS-CoV-2 N protein mAb at the optimal concentration was added to each well, and the microtitre plate was then gently taped for mixing, followed by a 1 h incubation at 37 °C. After washing with PBST and an incubation with 1:1000 goat anti-mouse IgG secondary antibody conjugated with HRP at 37 °C for 30 min, the plate was washed again and the enzymatic reaction was developed and stopped, and the optical densities of the resultant reaction mixtures were read as described above. If the test serum sample did not contain any anti-SARS-CoV-2 N protein antibody, the mouse anti-SARS-CoV-2 N protein mAb would bind to the coated recombinant SARS-CoV-2 N protein, resulting in strong colour development. However, if anti-SARS-CoV-2 N protein antibodies were present in the test serum sample due to COVID-19, the serum anti-SARS-CoV-2 N protein antibodies would compete with the mouse anti-SARS-CoV-2 N protein mAb for the coated recombinant SARS-CoV-2 N protein and inhibit binding by the mouse anti-SARS-CoV-2 N protein mAb, resulting in an inverse proportional development of colour signal. A conjugate control, composed of 50 μL of mouse anti-SARS-CoV-2 N protein mAb and 50 μL of sample dilution buffer, was included for the interpretation of the cELISA results, which were expressed as percent inhibition calculated using the formula:

Percent inhibition (PI, %) = 100% − [(OD_450_ of test serum sample/OD_450_ of conjugate control) × 100%].

The serum dilution at which there was the largest difference between the PI values of the positive and negative samples was determined as the optimal serum dilution for the assay.

#### 2.4.3. Determination of the cELISA Cut-Off Value

Using 74 serum samples from Panel 2 as described above, the PI cut-off value of the cELISA developed was determined as the mean PI values for these negative samples + 3.5 × standard deviations (SDs).

#### 2.4.4. Determination of Analytical Sensitivity of the cELISA

The analytical sensitivity of the cELISA was determined using serum samples in Panel 1, where the two positive sera were two-fold serially diluted from 1:2 to 1:512 in the negative sera of their respective host species.

#### 2.4.5. Cross Reactivities to Serum Samples Seropositive to βCoVs Other Than *Sarbecovirus* and Determination of Analytical Specificity of the cELISA

Using 49 serum samples from Panel 4 as described above, cross reactivities of the cELISA to serum samples seropositive to βCoVs other than *Sarbecovirus* were examined and the analytical specificity of the cELISA was determined.

#### 2.4.6. Preliminary Testing on Diagnostic Performance with Sera from Experimentally Infected Animals

Using 75 serum samples from Panels 2.1 and 3 as described above, the ability of the cut-off value determined above to distinguish infected and uninfected individuals, as well as the utility of the cELISA on different animals, were preliminarily examined.

### 2.5. Evaluation of the Diagnostic Performance of the cELISA

The diagnostic specificity of the cELISA was determined using 94 serum samples from Panel 5 as described above, whereas the diagnostic sensitivity of the cELISA was determined using 61 serum samples from Panel 6 as described above.

### 2.6. Statistical Analyses

Unless otherwise specified, all statistical analyses for all the data obtained from the in-house developed recombinant SARS-CoV-2 N protein-based indirect ELISA, the commercial indirect ELISA, as well as the cELISA developed in this study were performed using Prism 7.0.0 (GraphPad Software, San Diego, CA, USA). Best fit curves were generated by 4-parameter logistic regression, the Boltzmann sigmoid equation, or the one phase exponential decay equation. Sensitivities, specificities, and their confidence intervals were computed by the C.I. Calculator: Diagnostic Statistics [22].

## 3. Results

### 3.1. Experimental Production of Anti-SARS-CoV-2 Antisera by Laboratory Animal Immunization

**Figure 1:**
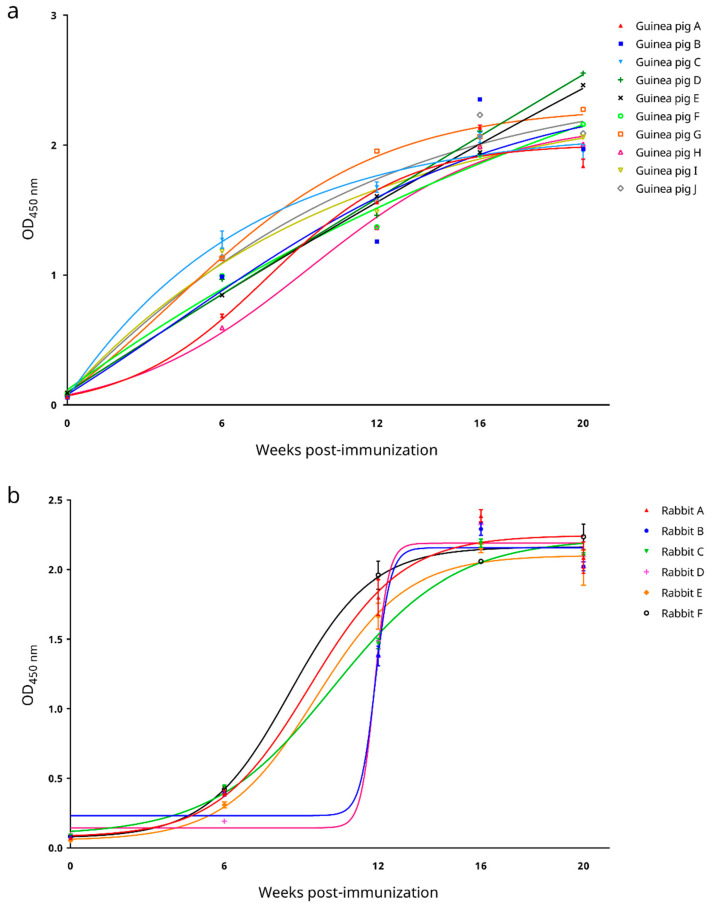
Anti-SARS-CoV-2 antibody responses in the laboratory animals post-inactive SARS-CoV-2 immunization as determined by an in-house developed recombinant SARS-CoV-2 nucleocapsid protein-based indirect enzyme-linked immunosorbent assay. (**a**) Guinea pigs and (**b**) rabbits. The best fit curves were generated by 4-parameter logistic regression. OD_450nm_, optical density at 450 nm.

Ten guinea pigs (A–J) and six rabbits (A–F) were immunized with inactive SARS-CoV-2 virions. All immunized animals developed immune responses and generated anti-SARS-CoV-2 antisera. Indirect ELISA based on the recombinant SARS-CoV-2 N protein showed that for both guinea pigs and rabbits, the serum level of anti-SARS-CoV-2 antibodies prior to immunization was insignificant (Figure 1). For guinea pigs, anti-SARS-CoV-2 antibodies were generated starting around 6 weeks post-immunization and their levels began to reach a plateau at 20 weeks post-immunization (Figure 1a); whereas for rabbits, antibody levels began to increase between 6–12 weeks post-immunization and reached a plateau at 16 weeks post-immunization (Figure 1b).

### 3.2. Reactivity and Specificity of Recombinant SARS-CoV-2 N Protein with Mouse Anti-SARS-CoV-2 N Protein mAb

**Figure 2:**
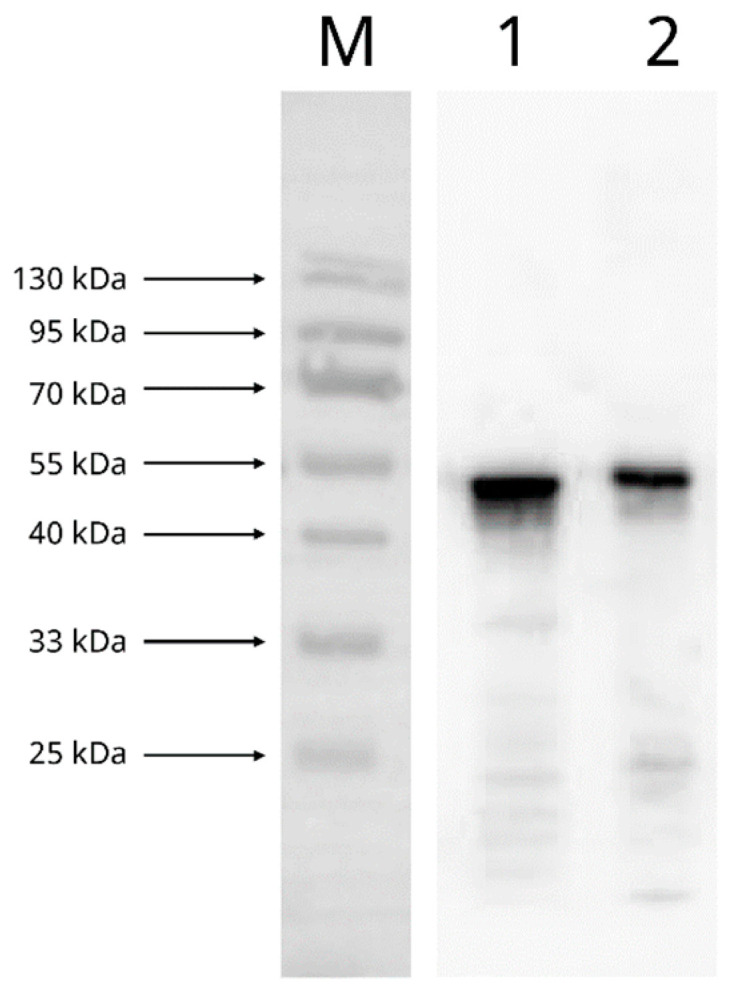
Western blot showing the reactivity between the recombinant SARS-CoV-2 nucleocapsid (N) protein and the mouse anti-SARS-CoV-2 N protein monoclonal antibody with inactive SARS-CoV-2 virion as the positive control. Lane M, protein marker; lane 1, inactive SARS-CoV-2 virion; lane 2, recombinant SARS-CoV-2 nucleocapsid protein.

**Figure 3:**
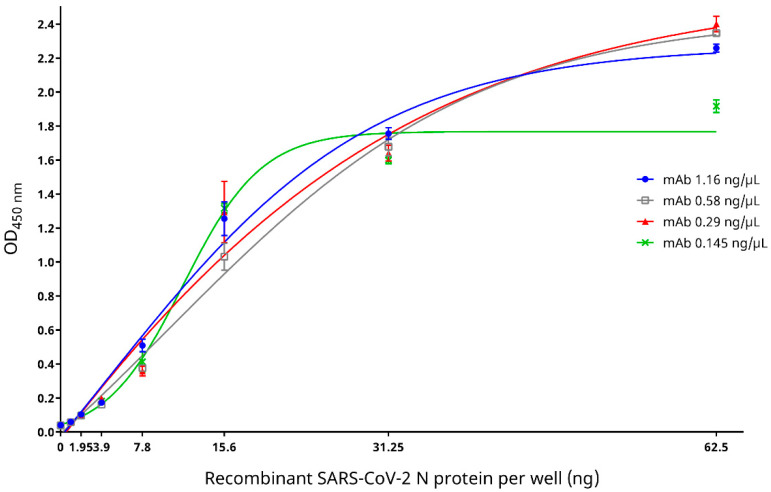
Specificity of the mouse anti-SARS-CoV-2 nucleocapsid (N) protein monoclonal antibody (mAb) at various concentrations to different amounts of the SARS-CoV-2 N protein as determined by an in-house developed recombinant SARS-CoV-2 N protein-based indirect enzyme-linked immunosorbent assay. The best fit curves were generated by the Boltzmann sigmoid equation. OD_450nm_, optical density at 450 nm.

Western blotting demonstrated strong reactivity between the recombinant SARS-CoV-2 N protein and the mouse anti-SARS-CoV-2 N protein mAb (Figure 2). Indirect ELISA based on the recombinant SARS-CoV-2 N protein showed that when mouse anti-SARS-CoV-2 N protein mAb concentrations of 0.29–1.16 ng/µL were used, the optical densities increased with an increasing amount of the recombinant SARS-CoV-2 N protein, which began to reach a plateau (OD_450nm_ ~2.2–2.4) at 62.5 ng of the recombinant SARS-CoV-2 N protein (Figure 3). On the other hand, when a mouse anti-SARS-CoV-2 N protein mAb concentration of 0.145 ng/µL was used, although the optical density also increased with an increasing amount of the recombinant SARS-CoV-2 N protein, it reached a lower plateau (OD_450nm_ ~1.8) at 31.25 ng of the recombinant SARS-CoV-2 N protein (Figure 3). Since the highest optical density was obtained when a mouse anti-SARS-CoV-2 N protein mAb concentration of 0.29 ng/µL and a recombinant SARS-CoV-2 N protein amount of 62.5 ng were used, this mAb/antigen combination was determined as the optimal condition for use in the cELISA.

### 3.3. Epitope Blocking and Determination of Optimal Serum Dilution for the cELISA

**Figure 4:**
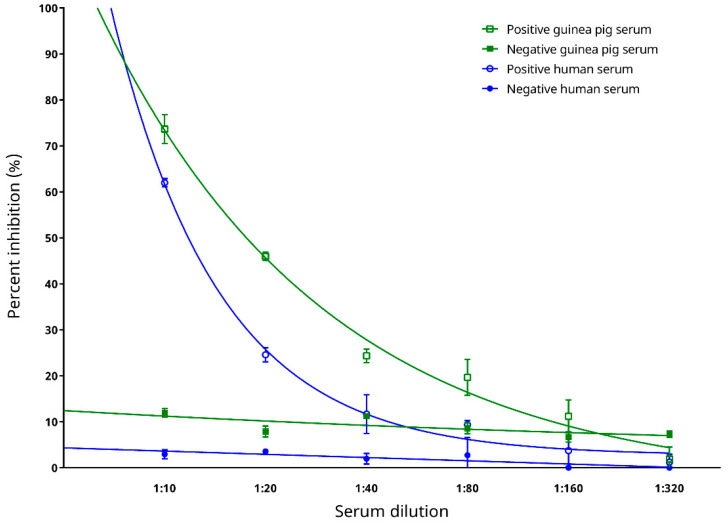
Effect of different serum dilutions on the in-house developed recombinant SARS-CoV-2 nucleocapsid (N) protein-based competitive enzyme-linked immunosorbent assay. The best fit curves were generated by the one phase exponential decay equation.

The binding of the mouse anti-SARS-CoV-2 N protein mAb to the coated antigen (recombinant SARS-CoV-2 N protein) was competitively inhibited in the presence of anti-SARS-CoV-2 antibodies in the positive serum samples (Figure 4). As the test sera became more diluted from 1:10 to 1:320, the PI also decreased accordingly, indicating that there was a lower concentration of anti-SARS-CoV-2 antibodies present which resulted in a decrease in competition with the mouse anti-SARS-CoV-2 N protein mAb for the coated antigen. The PI values of the positive guinea pig serum sample at dilutions of 1:10–1:160, and those of the positive human serum sample at dilutions of 1:10–1:40, remained more than two SDs above the means of the respectively negative serum samples (Figure 4). This indicated the ability of the cELISA to distinctly separate and determine a sample as SARS-CoV-2-positive or -negative when a serum dilution of 1:40 or lower was used. The largest difference between the PI values of the positive and negative samples was observed at the serum dilution of 1:10, and therefore, this serum dilution was selected for use in the subsequent development and evaluation of the cELISA.

### 3.4. Determination of the Cut-Off Value of the cELISA

**Figure 5:**
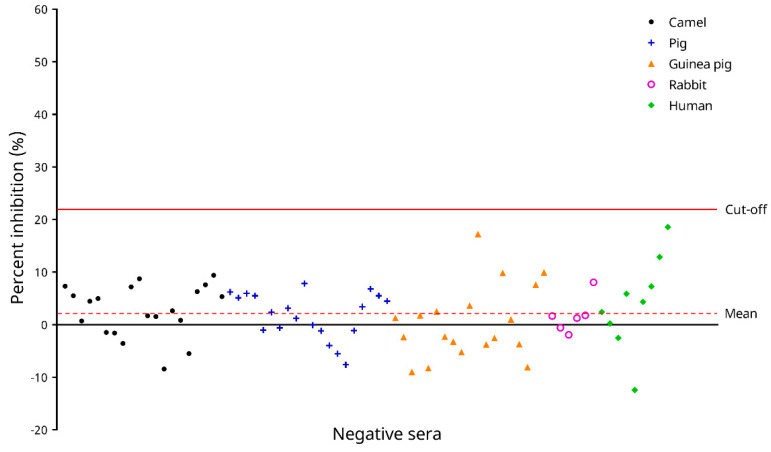
Determination of the cut-off percent inhibition (PI) value of the in-house developed recombinant SARS-CoV-2 nucleocapsid (N) protein-based competitive enzyme-linked immunosorbent assay (cELISA). Distribution of PI values for 74 negative serum samples from humans/animals free from COVID-19 as determined by the cELISA are shown. The cut-off PI value (22.345%) was determined as the mean PI values for the negative samples + 3.5 × standard deviations.

The PI cut-off value of the cELISA was determined using 74 negative serum samples from humans/animals free from COVID-19. For these samples, the PI values ranged from −12.432% to 18.574%, with a mean PI of 1.936% and an SD of 5.831% (Figure 5). Hence, the PI cut-off value was determined as 1.936% + 3.5 × 5.831% = 22.345%. Using this cut-off value, all negative samples possessed PI values below the cut-off (Figure 5).

### 3.5. Determination of Analytical Sensitivity

**Figure 6:**
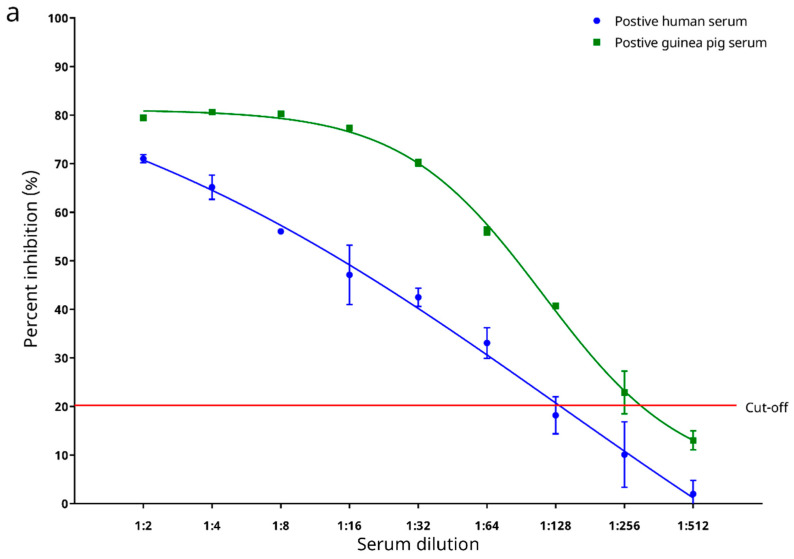

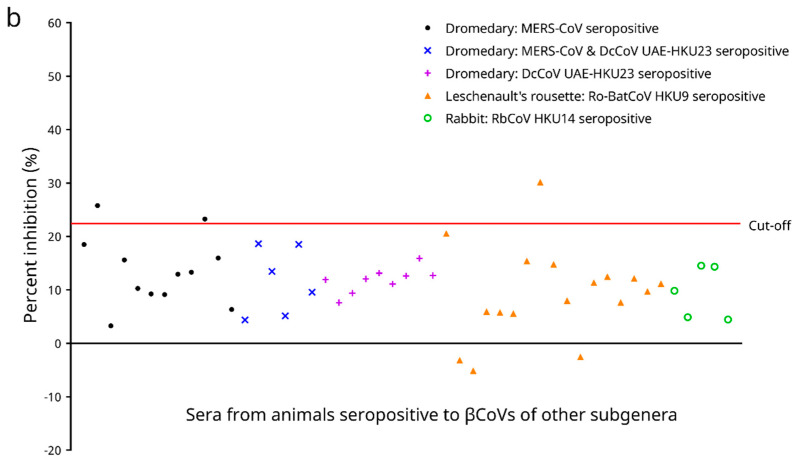
(**a**) Determination of analytical sensitivity of the in-house developed recombinant SARS-CoV-2 nucleocapsid (N) protein-based competitive enzyme-linked immunosorbent assay (cELISA). The best fit curves were generated by 4-parameter logistic regression. (**b**) Distribution of percent inhibition values for 49 serum samples from animals seropositive to betacoronaviruses (βCoVs) other than *Sarbecovirus*, including: dromedary (*Camelus dromedarius*) serum samples seropositive to *Middle East respiratory syndrome-related coronavirus* (MERS-CoV); dromedary serum samples seropositive to both MERS-CoV and dromedary camel coronavirus UAE-HKU23 (DcCoV UAE-HKU23); dromedary serum samples seropositive to DcCoV UAE-HKU23; Leschenault’s rousette (*Rousettus leschenaulti*) serum samples seropositive to *Rousettus bat coronavirus HKU9* (Ro-BatCoV HKU9); and rabbit (*Oryctolagus cuniculus*) serum samples seropositive to rabbit coronavirus HKU14 (RbCoV HKU14), as determined by the in-house developed recombinant SARS-CoV-2 nucleocapsid protein-based competitive enzyme-linked immunosorbent assay (cELISA).

Two positive and two negative sera from Panel 1 were used to test for the analytical sensitivity of the cELISA. The highest dilutions for specific detection of serum anti-SARS-CoV-2 antibodies were determined to be 1:64 for the positive human serum, and 1:256 for the positive guinea pig serum (Figure 6a) using the PI cut-off value of 22.345%.

### 3.6. Cross Reactivities to Serum Samples Seropositive to βCoVs Other Than Sarbecovirus and Analytical Specificity

Cross reactivities of the cELISA developed were assessed using 49 serum samples from animals that previously tested seropositive to βCoVs other than *Sarbecovirus*, including MERS-CoV, DcCoV UAE-HKU23, Ro-BatCoV HKU9, and RbCoV HKU14, and the analytical specificity of the cELISA was determined. For these 49 samples, the PI values ranged from −5.139% to 30.159%, with a mean PI value of 11.180% and an SD of 6.721% (Figure 6b). Among all the 49 samples tested, three (6.1%), including two MERS-CoV seropositive dromedary sera (2/12, 16.7%) and one Ro-BatCoV HKU9 seropositive Leschenault’s rousette serum (1/17, 5.9%), possessed PI values above the cut-off of 22.345% (Figure 6b). On the other hand, no cross reactivity was observed between the DcCoV UAE-HKU23 seropositive dromedary sera and the recombinant SARS-CoV-2 N protein, as well as between the RbCoV HKU14 seropositive rabbit sera and the recombinant SARS-CoV-2 N protein as tested by the cELISA (Figure 6b). This suggested an analytical specificity of 93.9% (95% C.I.: 87.2–100.6%) for the cELISA developed when serum samples seropositive to βCoVs other than *Sarbecovirus* were tested.

### 3.7. Preliminary Testing on Diagnostic Performance with Sera from Experimentally Infected Animals

**Figure 7:**
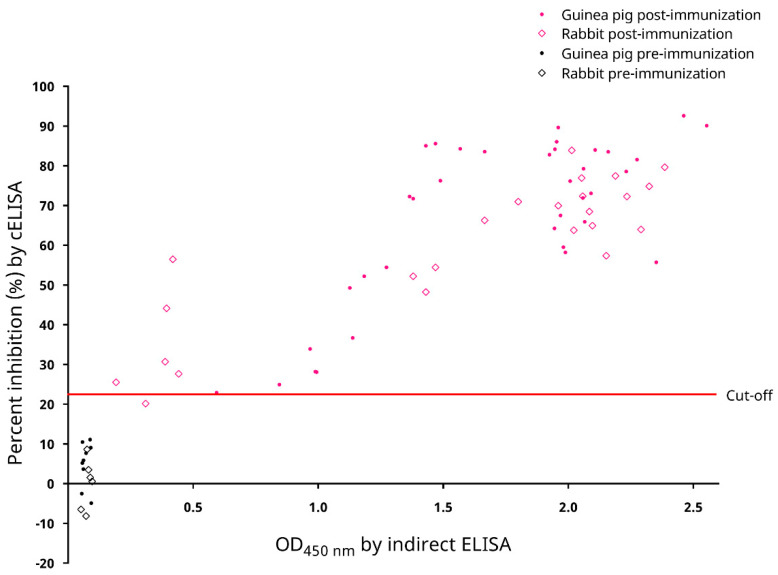
Distribution of percent inhibition (PI) values for 75 serum samples from laboratory animals pre- and post-immunization with the inactive SARS-CoV-2 virion as determined by the in-house developed recombinant SARS-CoV-2 nucleocapsid (N) protein-based competitive enzyme-linked immunosorbent assay (cELISA). The PI values were compared with their respective absorbance values determined by an in-house developed recombinant SARS-CoV-2 nucleocapsid protein-based indirect ELISA as shown in Figure 1. OD_450nm_, optical density at 450 nm.

Serum samples from the laboratory animals pre- and post-immunization with inactive SARS-CoV-2 virions were used to test for the preliminary diagnostic performance of the cELISA. For the 15 serum samples from pre-immunized animals, the PI values ranged from −8.159% to 11.096%, with a mean PI of 3.0162% and an SD of 6.216% (Figure 7). All pre-immunization samples possessed PI values below the cut-off. On the other hand, for the 60 serum samples from immunized animals, the PI values ranged from 20.148% to 92.620%, with a mean PI of 63.945% and an SD of 19.915% (Figure 7). One serum sample, from rabbit E 6 weeks post-immunization, possessed a PI value below the cut-off (Figure 7). Nonetheless, the cELISA was able to detect antibody responses against SARS-CoV-2 in different animal hosts.

### 3.8. Diagnostic Performance of the cELISA

**Figure 8:**
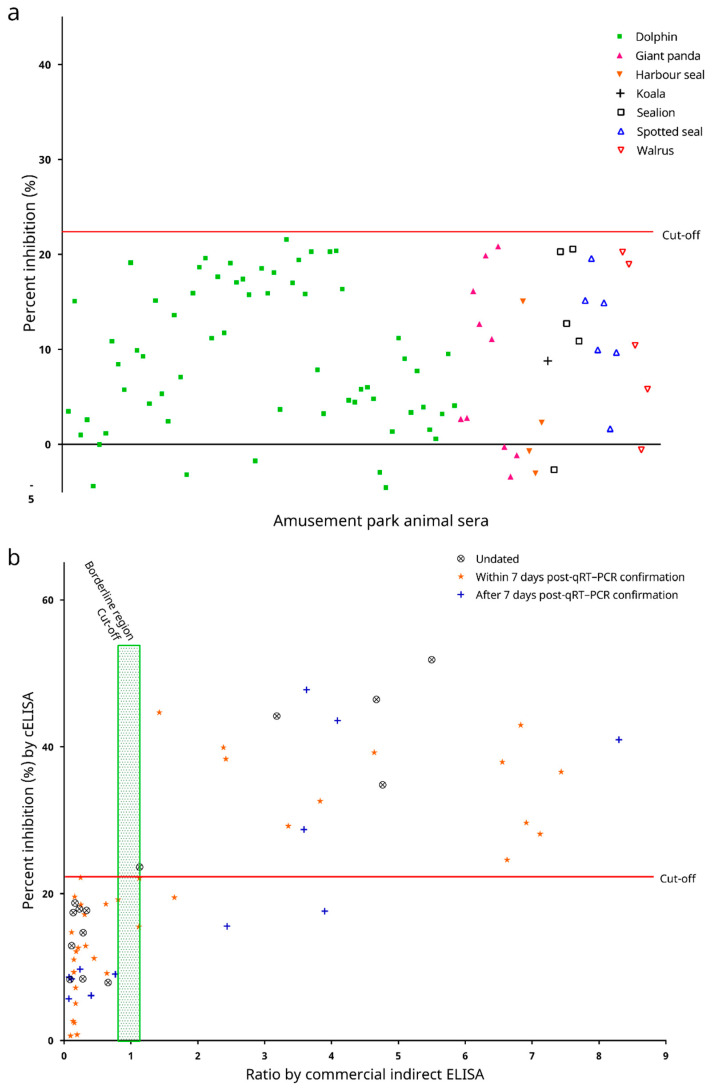
Distribution of percent inhibition (PI) values for (**a**) 94 serum samples from animals free from COVID-19 in an amusement park as determined by the in-house developed recombinant SARS-CoV-2 nucleocapsid protein-based competitive enzyme-linked immunosorbent assay (cELISA); and (**b**) 61 serum samples from laboratory confirmed human COVID-19 patients as determined by the cELISA, which were compared with their respective absorbance values determined by a commercial indirect ELISA kit.

When the cELISA was evaluated using 94 serum samples from animals free from COVID-19 from an amusement park, the PI values ranged from −4.556% to 21.590%, with a mean PI of 9.281% and an SD of 7.694%. Using the PI cut-off value of 22.345% determined above, all samples possessed PI values below the cut-off. Hence, the diagnostic specificity of the cELISA obtained using these amusement park animals’ sera was 100% (95% C.I.: 100–100%; Figure 8a).

Serum samples from laboratory confirmed human COVID-19 patients were also collected to evaluate the cELISA. These samples were tested for the presence of anti-SARS-CoV-2 antibodies using a commercial indirect ELISA kit. Among all the 61 samples analysed, 34 (55.7%) were determined as seronegative, 1 (1.6%) as borderline, and 26 (42.6%) as seropositive by the commercial indirect ELISA (Figure 8b). When the cELISA was evaluated using all these 61 samples, the PI values ranged from 0.637% to 51.845%, with a mean of 20.889% and an SD of 13.773%. Using the PI cut-off value of 22.345% as determined above, 40 samples (65.6%) were tested seronegative, and 21 samples (34.4%) were tested seropositive by the cELISA. When only serum samples tested seropositive (*n* = 26) by the commercial indirect ELISA were considered, the PI values obtained from the cELISA ranged from 15.485% to 51.845%, with a mean PI of 33.688% and an SD of 10.597%. Twenty-one samples also tested seropositive by the cELISA, although 5 samples were tested as false-negatives, indicating a diagnostic sensitivity of 80.8% (95% C.I.: 65.6–95.9%). On the other hand, when only serum tested seronegative (*n* = 34) by the commercial indirect ELISA were considered, the PI values obtained from the cELISA ranged from 0.637% to 22.184%, with a mean PI of 11.151% and an SD of 5.741%. All of these samples also tested seronegative by the cELISA, indicating a diagnostic specificity of 100% (95% C.I.: 100–100%; Figure 8b) as well. As for the serum sample falling inside the borderline region when tested by the commercial indirect ELISA, it was tested seronegative by the cELISA (Figure 8b). Overall, there is a strong positive correlation between the absorbance ratios as determined by the commercial indirect ELISA kit, and the PI values as determined by the cELISA (Pearson correlation coefficient r = 0.7530 [95% C.I.: 0.6185–0.8447], two-tailed *p* < 0.0001, and Spearman correlation coefficient r = 0.7825 [95% C.I.: 0.6566–0.8660], two-tailed *p* < 0.0001).

## 4. Discussion

A serodiagnosis for COVID-19 requires the detection of anti-SARS-CoV-2 antibodies in serum samples of infected animals. In general, host-specific secondary antibodies are needed if an ELISA is to be used for the detection of the presence of antibodies in a particular kind of animal. However, secondary antibodies may not be readily available for all animal species. For example, in our past experience in detecting the influenza virus in a giant panda from a local amusement park in 2019 and examining its corresponding immune response [23], we found that secondary antibodies against giant pandas were not commercially available. Therefore, the hemagglutinin inhibition test, instead of the more specific serological tests, had to be used. However, such non-specific serological tests only exist for a few types of infections. For other infectious diseases, such as COVID-19, for which non-specific serological tests are lacking, host-specific secondary antibodies would need to be generated. For instance, in our previous attempt to develop an ELISA-based serodiagnostic assay for aspergillosis in falcons (*Falco* spp.), we produced a polyclonal anti-falcon immunoglobulin Y (IgY) antibody by immunzing laboratory guinea pigs with purified falcon IgY, followed by guinea pig IgG purification and HRP labelling. This was because anti-falcon IgY antibodies were not available in the market [24]. In addition, for infections affecting multiple animal hosts, secondary antibodies against all animal species concerned would be necessary for their serodiagnosis. A high diagnostic cost would then be associated with the purchasing and/or generation of many different kinds of secondary antibodies for these infectious diseases. In order to solve these problems, the use of the cELISA would become a feasible approach since, instead of measuring the amount of serum antibodies from the animal hosts, the detection antibody in the cELISA recognizes the monoclonal antibody which competes with host serum antibodies for the coated antigen. Therefore, the cELISA system only measures the amount of monoclonal antibody bound and thus only one secondary antibody against the monoclonal antibody, generated from one single animal species, is required. As an illustration, we recently developed a cELISA for the serodiagnosis of glanders [25], a highly communicable disease caused by the bacterium *Burkholderia mallei* affecting a variety of animals [26]. Since a mouse monoclonal antibody is used in this assay, only a secondary antibody against mouse IgG is needed for antibody detection [25]. Similarly, in the present study, we developed a cELISA-based serological test which can serve to detect anti-SARS-CoV-2 antibodies across many different animal species.

The recombinant SARS-CoV-2 N protein-based cELISA developed here is sensitive and specific for the serodiagnosis of COVID-19. The N proteins of CoVs are abundantly expressed during infection and are highly immunogenic [27]. They, along with the spike (S) proteins, are popular antigens used for serodiagnosis of CoV infections [27]. In our previous experience, when indirect ELISAs were developed for severe acute respiratory syndrome coronavirus (SARS-CoV), we observed that the SARS-CoV N protein-based IgG ELISA possessed a significantly higher sensitivity than the S protein-based IgG ELISA [28]. For SARS-CoV-2, a recent serological study using a luciferase immunoprecipitation system assay also suggested that measurement of anti-SARS-CoV-2 N protein antibodies may be more sensitive than measuring anti-SARS-CoV-2 S protein antibodies for detecting early infection in humans [29]. For the present study, a recombinant SARS-CoV-2 N protein was used as the coating antigen to develop a cELISA. This cELISA allows the detection of anti-SARS-CoV-2 antibodies in animal sera through competition with a mouse anti-SARS-CoV-2 N protein mAb to interact with the coated antigen. Assessment of the analytical performance of this cELISA showed that it possessed a high analytical sensitivity, with limits of detection down to 1:64 and 1:256 as the last reactive dilutions of the positive human and guinea pig sera, respectively (Figure 6a). The cELISA was also analytically specific (93.9% [95% C.I.: 87.2–100.6%]). In our previously developed recombinant N protein-based ELISA for SARS-CoV [30], cross reactivity was observed between the recombinant SARS-CoV N protein and 14.3% of convalescent human patient serum samples, which were seropositive to human coronavirus OC43 (HCoV-OC43, species *Betacoronavirus 1*, subgenus *Embecovirus*) [31]. On the contrary, the recombinant SARS-CoV-2 N protein used in the cELISA developed in this study did not cross react with any serum sample seropositive to DcCoV UAE-HKU23 or RbCoV HKU14, both of which are also members of the subgenus *Embecovirus* (Figure 6b). However, it was of note that in our experiment two out of the twelve MERS-CoV (subgenus *Merbecovirus*) seropositive dromedary sera, and one out of the seventeen Ro-BatCoV HKU9 (subgenus *Nobecovirus*) seropositive Leschenault’s rousette sera still cross reacted (Figure 6b). Preliminary assessment of the diagnostic performance of the cELISA showed that it could well distinguish serum samples from laboratory animals pre- and post-immunization with inactive SARS-CoV-2 virions, except for one rabbit serum sample collected 6 weeks post-immunization (rabbit E, Figure 7). The low PI value for this serum sample was likely due to the fact that this rabbit had not yet seroconverted at this early timepoint, which was also reflected by the low absorbance measured for this sample through the indirect ELISA (Figure 7). When the diagnostic performance of the cELISA was evaluated using sera from COVID-19-free animals as well as sera from qRT–PCR-confirmed COVID-19 human patients, a 100% diagnostic specificity (Figure 8b) and an 80.8% (95% C.I.: 65.6–95.9%) diagnostic sensitivity (Figure 8b) was demonstrated. Notably, the PI values obtained for the sera from qRT–PCR-confirmed COVID-19 human patients possessed a strong positive correlation with their respective absorbance ratios determined by the commercial indirect ELISA (Figure 8b).

Given its sensitive and specific diagnostic performance with minimal cross reactivities to antisera against βCoVs other than *Sarbecovirus*, the cELISA developed in this study is a promising serodiagnostic tool for COVID-19 in animals. The current cELISA can be used for testing virtually all kinds of animals. This is because the detection antibody in the assay recognises the amount of monoclonal antibody bound to the coating antigen instead of serum antibodies from the animals and so the assay is not specific to a particular kind of animal. Therefore, this cELISA could serve the purpose of a collection of many normal ELISAs which are specifically designed for testing only one particular animal. There are also a number of advantages in employing this cELISA over nucleic acid amplification tests such as qRT–PCR or reverse transcription loop-mediated isothermal amplification (RT–LAMP) [32] as used in diagnosing COVID-19 in humans. First, serological assays are cheap and they do not require special facilities, instruments, or expertise which may not be readily available in resource limited countries [33]. Second, this cELISA is an antibody detection assay, and therefore it checks for any seroconversion of the animals and could confirm whether the animals are genuinely infected by the virus or not. Third, as opposed to obtaining respiratory specimens from animals for subsequent nucleic acid extraction, blood sampling for animals is easier to perform and not as dangerous. In addition to serodiagnosing individual animals, this cELISA can also be used for the mass screening of COVID-19 in husbandry animals. Animal farms and zoos usually house a large quantity of animals belonging to many different species, and so this cELISA is particularly useful in these settings. Notably, the cELISA can be incorporated into routine blood testing in animal farms or zoos, and therefore, additional specimen sampling is not required. Furthermore, a small yet representative number of animals from an independent herd can be sampled for testing to infer the immune status of animals in the entire herd, without the need of testing every single individual animal. When a high percentage of animals in a population is tested positive by this cELISA, they should be isolated and have a nucleic acid amplification test performed to confirm the occurrence of any ongoing outbreak. For example, mink farmers could collect blood samples from their animals and submit for testing by this cELISA at regular intervals to monitor any COVID-19 infection in their minks. While any positive result could help mink farmers identify infected animals/herds and initiate isolation or mass slaughter strategies, negative results from such routine testing could help avoid unnecessary loss of animal lives.

## Data Availability

The data presented in this study are available on request from the corresponding authors.
